# Effectiveness of Ozone Treatment, Ultrasonic Treatment, and Ultraviolet Irradiation in Removing *Candida albicans* Adhered to Acrylic Resins Fabricated by Different Manufacturing Methods

**DOI:** 10.3390/ma19010053

**Published:** 2025-12-23

**Authors:** Chihiro Kaneko, Tomofumi Sawada, Taichi Ishikawa, Toshitaka Miura, Takuya Kobayashi, Shinji Takemoto

**Affiliations:** 1 Division of Removable Prosthodontics and Oral Rehabilitation, Department of Prosthodontics, School of Dentistry, Iwate Medical University, 19-1 Uchimaru, Morioka 020-8505, Iwate, Japan; kanechi@iwate-med.ac.jp (C.K.); kobataku@iwate-med.ac.jp (T.K.); 2 Department of Biomedical Engineering, Iwate Medical University, 1-1-1 Idaidori, Yahaba-cho, Shiwa-gun 028-3694, Iwate, Japan; sawada@dent.asahi-u.ac.jp; 3 Department of Dental Material Science, Division of Oral Functional Science and Rehabilitation, Asahi University School of Dentistry, Hozumi 1851, Mizuho 501-0296, Gifu, Japan; 4 Division of Molecular Microbiology, Department of Microbiology, Iwate Medical University, 1-1-1 Idaidori, Yahaba-cho, Shiwa-gun 028-3694, Iwate, Japan; tishikaw@iwate-med.ac.jp (T.I.); tosmiura@iwate-med.ac.jp (T.M.)

**Keywords:** denture cleaning method, ozonated water, ultrasonic cleaning, ultraviolet irradiation, *Candida albicans*, acrylic resins, additive manufacturing

## Abstract

Acrylic resins are commonly used for denture bases due to ease of molding but are prone to water absorption and microbial contamination. This study aimed to evaluate the effects of ozonated water immersion (OZ), ultrasonic cleaning (US), and ultraviolet (UV) irradiation on the removal of *Candida albicans* from acrylic resins produced by heat curing and additive manufacturing. The resin specimens were then subjected to treatment with OZ, US, UV irradiation, and commercial denture cleansers. Following treatment, the number of viable *C. albicans* cells was quantified and statistically analyzed (α = 0.05), morphology was observed under a scanning electron microscope (SEM) and fluorescence imaging. OZ, US, and UV irradiation significantly reduced the viable *C. albicans* count. Notably, the combination of the three treatments achieved a reduction exceeding 99.9% of viable cells. Although SEM revealed that *C. albicans* remained on the specimens, fluorescence imaging demonstrated a progressive decrease in viable cells and an increase in dead cells with each treatment, with the greatest effect observed when the three treatments were combined. The difference of removal behaviors of *C. albicans* among fabrication methods was not observed, comparable to denture cleaners. The combined application of all three treatments was the most effective strategy for microbial removal.

## 1. Introduction

The global elderly population is increasing [1], leading to a projected rise in the proportion of denture wearers due to age-related tooth loss [2]. Removable dentures are widely regarded as a practical prosthetic option, as they are minimally invasive and can restore masticatory function without imposing excessive burden on elders. A removable denture consists of a base, including the framework and retention components and artificial teeth. Base materials may consist of resins, such as acrylic resin, or metals, including cobalt-chromium alloys. When placed in the oral cavity, these materials are exposed to harsh environments, including temperature and pH changes and microbial contamination. Among these, acrylic resins are commonly used for denture bases due to their low cost. However, they are highly water-absorbent and susceptible to microbial colonization [3,4,5].

Acrylic resins used for denture bases are classified as heat-polymerizable (HP), autopolymerizable, and thermoplastic and can also be fabricated using subtractive or additive manufacturing (AM) methods within computer-aided design (CAD) and computer-aided manufacturing (CAM) systems, reflecting advances in digital technology. In heat-polymerized resins, heating above 60 °C induces the decomposition of benzoic peroxide, generating free radicals that initiate polymerization of methyl methacrylate. In contrast, additive manufacturing employs photo-activated resin monomers, which are polymerized using vat photopolymerization (VAT) or material jetting techniques. Although additive manufacturing allows for the production of more complex denture geometries compared with conventional heat-polymerized methods, challenges remain, including incomplete post-polymerization and the presence of residual monomers.

Microbial adhesion to dentures is influenced by the surface properties of the denture material and the salivary pellicle [6,7,8]. Microorganisms colonize surface irregularities of denture bases and form biofilms [9]. Denture plaques frequently harbor pathogenic microorganisms, including bacteria associated with caries and periodontal disease, respiratory pathogens, and gastrointestinal infectious agents [9,10,11,12,13]. Dentures serve as reservoirs of microorganisms for the pharynx [14] and have been implicated in the development of local and systemic conditions, such as denture stomatitis and aspiration pneumonia [15,16]. *Candida albicans* specifically adheres to acrylic resin surfaces in association with surface roughness [17]. *C. albicans* is a dimorphic fungus commonly found in the oral cavity, skin, gastrointestinal tract, and reproductive organs [18]. Its detection rate in the oral cavity is higher among denture wearers. As *C. albicans* serves as a scaffold for biofilm formation with other microorganisms [19,20,21], its efficient removal from denture surfaces is essential. Maintaining proper oral hygiene and ensuring the cleanliness of dentures are essential for preserving the health of denture wearers. However, inadequate denture cleaning is commonly observed among elders due to reduced manual dexterity and other factors. Furthermore, in nursing home settings, the responsibility of cleaning dentures places a considerable burden on caregivers.

Denture cleaning methods are generally classified as mechanical or chemical cleanings [3,22]. Mechanical cleaning involves the physical removal of biofilm, typically using denture brushes or ultrasonic devices [23,24]. Chemical cleaning employs denture cleansing agents containing peroxides, enzymes, hypochlorite, or other acidic compounds to achieve microbial removal [25,26,27]. In practice, a combination of mechanical and chemical cleaning is recommended to maintain denture hygiene [22,28,29]. Despite numerous studies evaluating the effectiveness of these cleaning approaches, complete elimination of microorganisms from denture base materials remains difficult and a problem [30,31,32,33].

The sterilizing effects of ozone and ultraviolet (UV) light have been utilized in various fields, including environmental hygiene, food safety, and medicine. Ozonated water exhibits strong oxidative properties and is considered safe due to its lack of persistence. In vitro studies have demonstrated that ozonated water and UV irradiation can reduce the number and growth of *C. albicans* on heat-cured acrylic resin specimens [34,35,36]. However, a few studies have comprehensively investigated their effectiveness in denture cleaning, particularly with respect to microbial removal.

In this study, the effects of immersion in ozonated water, ultrasonic cleaning, and UV irradiation on *C. albicans* adhering to denture base acrylic resin were evaluated, with the aim of establishing a simple and safe method for denture cleaning. The first null hypothesis was that no significant difference would be observed in the removal of *C. albicans* from acrylic resins fabricated by heat curing following immersion in ozonated water, ultrasonic cleaning, and UV irradiation, and the second was also no significant difference of removal *C. albicans* on resin materials fabricated by additive manufacturing among three surface treatments.

## 2. Materials and Methods

### 2.1. Fabrication of Resin Specimens

Resin specimens for fungal adhesion tests were prepared using heat curing and additive manufacturing methods. For the heat-cured acrylic resin specimens (Acron^®^, livepink, GC Co, Tokyo, Japan), powder and liquid components were mixed according to standard procedures. The mixture was placed into a Teflon mold (15.0 mm × 72.0 mm × 2.5 mm) and heat cured to produce a plate-shaped resin. The plates were subsequently cut into specimens with a size of 14.0 mm × 14.0 mm × 2.0 mm (*n* = 110).

Additive manufactured resin specimens were produced using the cara Print 4.0 Pro (Kulzer Japan Co., Ltd., Tokyo, Japan) system with dima Print Denture Base (Kulzer Japan Co., Ltd., Tokyo, Japan) as the fabrication material. The printing process employed a wavelength of 385 nm and a stacking pitch of 100 μm. Post-curing was performed using a care Print LEDcure unit (Kulzer Japan Co., Ltd., Tokyo, Japan) at an irradiation intensity of 100% and a chamber temperature of 60 °C under glycerin immersion for 15 min to achieve complete polymerization. During post-curing with the cara LED cure unit, the total irradiation power applied was 150 W. Resin specimens were initially fabricated at dimensions of 15.8 mm × 15.8 mm × 3.0 mm and then adjusted to 14.0 mm × 14.0 mm × 2.0 mm (*n* = 40).

The resin specimens were sterilized by steam autoclaving at 121 °C for 15 min prior to use (Hiclave HG-50, HIRAYAMA Manufacturing Co., Saitama, Japan).

### 2.2. Preparation of Fungal Strains, Suspensions, and Adhesion Models

The *C. albicans* SC5314 strain was used in this study. The strain was pre-cultured in yeast extract peptone dextrose (YPD, BD Difco TM YPD Broth, Franklin Lakes, NJ, USA) liquid medium under aerobic conditions at 37 °C for 24 h. Following centrifugation (3000× *g*, 4 °C, 10 min), the supernatant was discarded. The pellets were resuspended in fresh YPD medium. The turbidity of the resulting suspension was adjusted to 2.0 at wavelength of 600 nm, corresponding to approximately 1.0 × 10^7^ CFU/mL, to maintain a consistent microbial concentration.

Sterilized resin specimens were placed in 6-well plates, and 100 μL of the *C. albicans* suspension was added to each well. To prevent desiccation and simulate the moist environment of the oral cavity, a dental cotton roll soaked with 3 mL of phosphate-buffered saline (PBS, Shimadzu Diagnostics Co., Tokyo, Japan) was placed in an empty well. The specimens were incubated under aerobic conditions at 37 °C for 24 h. After incubation, the resin specimens were transferred to new 6-well plates containing 1 mL of PBS per well. The edges of the resin specimens were held with tweezers, and 10 gentle movements were performed within the wells to remove weakly adherent *C. albicans*. The PBS-washed resin specimens were subsequently used as the adhesion model.

### 2.3. Treatments for the Adhesion Model (Table 1 and Figure 1)

The adhesion models were divided into 11 groups to evaluate the effects of individual and combined cleaning methods.

1. Immersion in tap water

Four milliliters of tap water were added to a 50 mL plastic tube, and the specimen of the adhesion model was immersed for 5 min in room temperature (Ctrl).

2. Immersion in ozonated water

Ozonated water was generated using an ozone buster (Earth Walker Trading Co., Ltd., Yamaguchi, Japan). The device was fully submerged in 1 L of tap water for 5 min. After ozone generation, the device was powered off, and 4 mL of the ozonated water was transferred to a 50 mL plastic tube, and the specimen of the adhesion model was immersed for 5 min (OZ). The concentration of ozonated water was measured using a dissolved ozone checker (DOC-05A, EBARAJITSUGYO Co., Ltd., Tokyo, Japan), and the concentration used in this study was 1.4 ± 0.3 ppm.

3. Ultrasonic cleaning

Four milliliters of tap water was added to a 50 mL plastic tube, and the specimens of the adhesion model were sonicated for 5 min by ultrasonic cleaner (US: UT-205HS, Sharp Co., Osaka, Japan).

4. UV irradiation

Adhesion model specimens were placed in a glass Petri dish and pouring 3 mL of PBS with the *C. albicans*-adhered surface facing upward. The specimens were irradiated with 285 nm LED UV light (PearlLab Beam, Nikkiso, Tokyo, Japan) at a distance of 40 mm. Irradiation duration times was applied for 1, 3, and 5 min (donated as UV1, UV3, and UV5, respectively). UV wavelength was at 285 nm and the irradiation distance of 70 mm, and the effective irradiance was 0.295 mW/cm^2^.

5. Immersion in denture cleaner

The specimens of adhesion model were immersed in 4 mL of tap water (40 °C) containing either peroxide (PO: Polident^®^, Haleon Japan K.K., Tokyo, Japan) or enzyme-based denture cleaner (PK: Pika (Blue)^®^, SHOFU INC., Kyoto, Japan) for 5 min.

6. Combination

Three combinations of OZ, US, and UV irradiation (UV1, UV3, or UV5) were applied to the specimens of adhesion models (TP1, TP3, TP5). Ultrasonic cleaning was performed by ultrasonic cleaners (UT-205HS, Sharp Co., Osaka, Japan and Multi ultrasonic cleaner SUC-100, SHOFU INC., Kyoto, Japan). All treatments were performed on heat-cured resin specimens, whereas Ctrl, UV3, peroxide denture cleaner (PK), and TP3 were performed on additive manufactured specimens. We prepared 10 specimens for each heat-cured resin specimen (11 groups) and for each additive manufactured resin specimen (4 groups), which were evaluated to viable *C. albicans* count: *n* = 6, SEM observation: *n* = 2, and fluorescence observation: *n* = 2.

After each treatment, the specimens were placed on 6-well plates, and poured to 1 mL of PBS per well. The edges of the specimens were held with tweezers and moved back and forth 10 times in the wells. This procedure was repeated three times to remove weakly adherent *C. albicans*.

**Figure 1:**
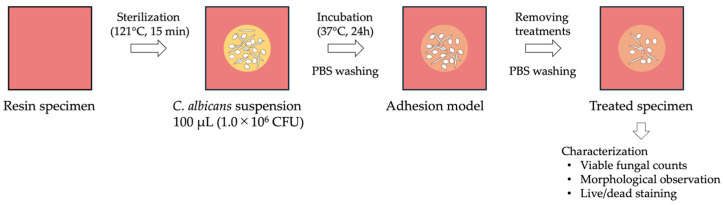
Flowchart illustrating the presentation of the adhesion model.

**Table 1:** Specimen code and the removing treatment methods.

Code	Treatment Methods
Ctrl	Immersion in tap water for 5 min
OZ	Immersion in ozonated water for 5 min
US	Ultrasonic cleaning for 5 min
UV1	UV (285 nm) irradiation for 1 min (distance 40 mm)
UV3	UV (285 nm) irradiation for 3 min (distance 40 mm)
UV5	UV (285 nm) irradiation for 5 min (distance 40 mm)
PO	Immersion in peroxide-type denture cleanser for 5 min
PK	Immersion in enzyme-type denture cleanser for 5 min
TP1	Immersion in ozonated water for 5 min, and ultrasonic cleaning for 5 min, and UV irradiation for 1 min
TP3	Immersion in ozonated water for 5 min, and ultrasonic cleaning for 5 min, and UV irradiation for 3 min
TP5	Immersion in ozonated water for 5 min, and ultrasonic cleaning for 5 min, and UV irradiation for 5 min

### 2.4. Evaluation of C. albicans Adhering to Resin Specimens

1. Viable fungal counts (CFU/mL)

Washed resin specimens were transferred to sterile centrifuge tubes containing 5 mL of PBS, and adherent *C. albicans* were collected by vortex mixing. A 10-fold serial dilution of the suspension was prepared using PBS, and aliquots of each dilution were plated onto YPD agar. The plates were incubated at 37 °C for 24 h under aerobic conditions. Viable fungal counts (CFU/mL) were determined by enumerating the colonies formed on the agar, with each colony representing a viable *C. albicans*.

2. Scanning electron microscopy observation (SEM)

After washing, the specimens of adhesion models and of all treatment groups were fixed in 2.5% glutaraldehyde at 4 °C for 2 h. Specimens were then dehydrated through a graded ethanol series (50%, 60%, 70%, 80%, 90%, and 100%) for each 30 min. Subsequently, the specimens were immersed in a mixture (1:1) of 100% ethanol and 2-methyl-2-propanol for 30 min at room temperature, followed by immersion in 2-methyl-2-propanol alone and freezing. The frozen specimens were transferred to a lyophilizer (VFD-21, Vacuum Device Inc., Ibaraki, Japan) to sublimate the 2-methyl-2-propanol. Finally, the resin specimens were coated with osmium using an osmium plasma coater (OPC60A, Filgen, Inc., Aichi, Japan) and observed under a scanning electron microscope (SEM) at an acceleration voltage of 2.0 kV.

3. Fluorescence observation of live and dead *C. albicans*

The resin specimens were fluorescently stained to evaluate the effects of each treatment on *C. albicans* viability. The LIVE/DEAD BacLight Bacterial Viability Kit (L7012, Thermo Fisher Scientific, Waltham, MA, USA) was used as the fluorescent staining reagent. The kit contained a green fluorescent nucleic acid stain (SYTO9) and a red fluorescent nucleic acid stain (propidium iodide: PI). Dye solutions were prepared by mixing 1.5 µL each of SYTO9 and PI per 1 mL of sterile PBS. The treated resin specimens were immersed in 1.5 mL of dye solution in 12-well plates and incubated in the dark for 15 min. After staining, the specimens were washed with 1 mL of PBS and observed under an all-in-one fluorescence microscope (BZ-9000, KEYENCE, Osaka, Japan). The excitation and emission wavelengths were set at 480/500 nm for SYTO9 and 535/635 nm for PI.

4. Statistical analysis

Viable fungal counts for each treatment of the specimens in heat-cured polymerizing and additive manufacturing were analyzed using Welch’s one-way analysis of variance, followed by Tukey’s multiple comparison test (BellCurve for Excel, Social Survey Research Information, Tokyo, Japan). The significant level was set to 0.05.

## 3. Results

### 3.1. Viable Counts of C. albicans

**Figure 2:**
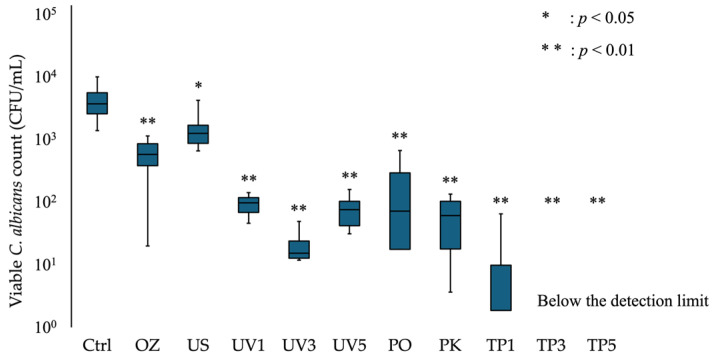
Viable *C. albicans* count adhering to heat-cured resin specimens after each treatment. The box-and-whisker plot illustrates the number of viable *C. albicans* in each treatment group. The boxes represent the interquartile range (first to third quartiles), and the horizontal line within each box indicates the median value. Asterisks (* and **) denote significant differences; * *p* < 0.05 and ** *p* < 0.01.

**Figure 3:**
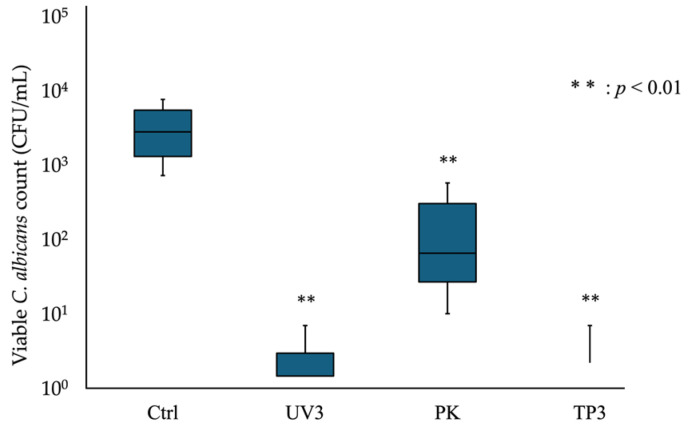
Viable *C. albicans* count adhering to the additive manufactured resin specimens after each treatment. The box-and-whisker plot illustrates the number of viable *C. albicans* in each treatment group. The boxes represent the interquartile range (first through third quartiles), and the horizontal line within each box indicates the median value. The asterisk (**) indicates a significant difference; ** *p* < 0.01.

For heat-cured resin specimens, the viable fungal count was 4.4 × 10^3^ CFU/mL in the Ctrl group and below the detection limit in the TP3 and TP5 groups (Figure 2). The viable count for the US group was significantly lower than that for the Ctrl group (*p* < 0.05), and another treatment group was also shown to be significantly lower (*p* < 0.01). For the additive manufactured resin specimens, viable *C. albicans* was detected in all treatment groups (Figure 3). The viable fungal counts were 3.2 × 10^3^ CFU/mL in the Ctrl group and 1.7 × 10^0^ CFU/mL in the TP3 group. Significant reductions in viable fungal counts were observed in the UV3, PK, and TP3 groups compared to Ctrl group (*p* < 0.01). The removal effect of each treatment was comparable between the heat-cured and additive manufactured resin specimens. The viable fungal count decreased with the combination of treatments, and the TP3 group demonstrated a similar reduction to that observed in the PK group.

### 3.2. SEM Observation

**Figure 4:**
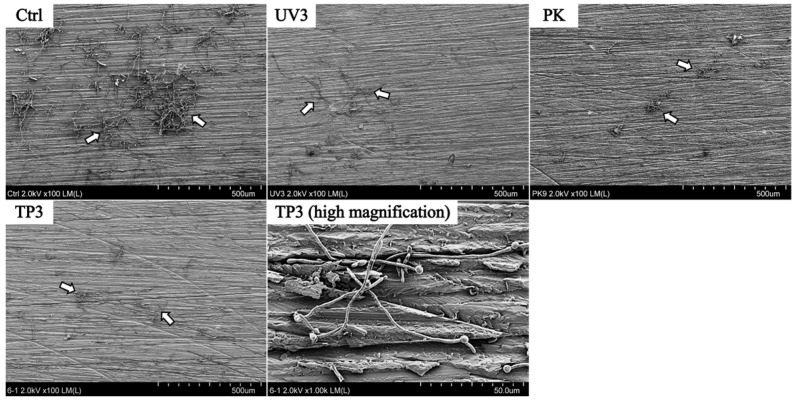
SEM images of heat-cured resin specimens after Ctrl, UV3, PK, TP3. White arrows indicate *C. albicans*.

SEM images of Ctrl, UV3, PK, and TP3 groups on the heat-cured resin specimens are shown in Figure 4. The Ctrl specimen was observed in *C. albicans* yeast and mycelial forms, with some appearing agglomerated. All specimens after treatment including OZ, US, and PO groups were also observed to have residual adhesion *C. albicans* on the surface, but the numbers were decreased.

**Figure 5:**
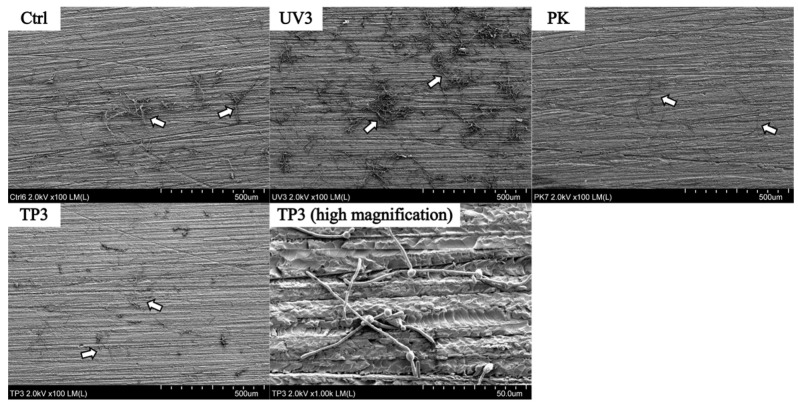
SEM images of additive manufactured resin specimens after Ctrl, UV3, PK, TP3. White arrows indicate *C. albicans*.

Similar to the heat-cured resin specimens, *C. albicans* remained on the surface of the additive manufactured resin specimens (Figure 5). The fungal on additive manufactured specimens after treatment were observed sparsely. However, the specimen showed the localized areas of *C. albicans* aggregation, as seen in the UV3 specimen.

### 3.3. Fluorescence Observation of Viable and Dead C. albicans

**Figure 6:**
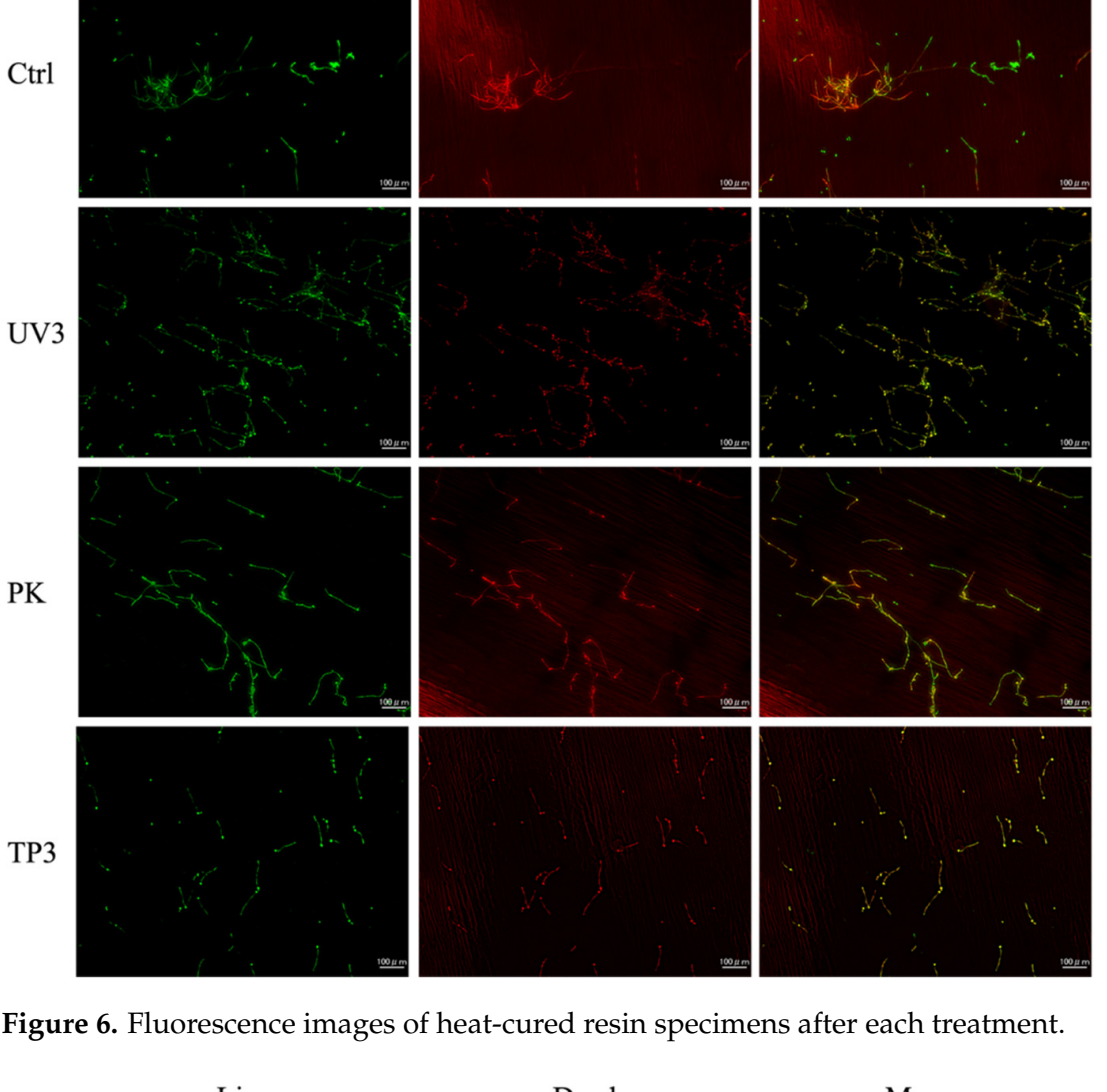
Fluorescence images of heat-cured resin specimens after each treatment.

**Figure 7:**
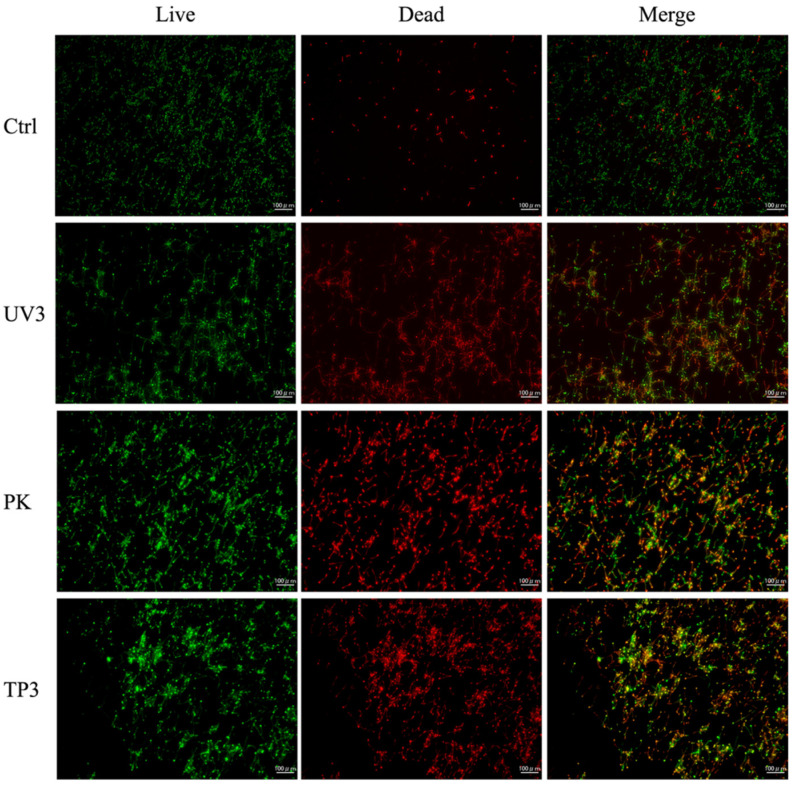
Fluorescence images of additive manufactured resin specimens after each treatment.

Fluorescence images of Ctrl, UV3, PK, and TP3 are presented (Figure 6 and Figure 7). Green fluorescence indicates viable *C. albicans*, whereas red fluorescence indicates dead *C. albicans*. In the heat-cured resin specimens, most *C. albicans* in the Ctrl group remained viable. The UV3, PK, and TP3 specimens showed the number of dead *C. albicans* increased, although some viable *C. albicans* were still observed. A similar trend was observed in the additive manufactured specimens, with viable *C. albicans* predominating in the Ctrl group. Conversely, the number of dead *C. albicans* tended to increase in UV3, PK, and TP3.

## 4. Discussion

In this study, the effects of ozonated water immersion, ultrasonic cleaning, and UV irradiation on the viability and persistence of *C. albicans* adhering to acrylic resin used for denture bases were examined. Acrylic resin bases were fabricated using the conventional heat curing method and the additive manufacturing method to investigate the effects of fabrication techniques on the adhesion and removal properties of *C. albicans*.

The advancement of digital denture technology has enabled the fabrication of dentures through both additive (AM) and subtractive (SM) manufacturing methods. In heat-cured resins, benzoyl peroxide is thermally decomposed to generate free radicals that initiate polymerization of the monomers during curing [4]. In contrast, dentures fabricated via additive manufacturing employ the vat photopolymerization method, in which a photosensitizer is incorporated into the resin monomer and polymerization is induced by light irradiation [37]. Although denture base materials in both methods can comply with the requirements of ISO 20795-1:2013 [38], appropriate cleaning protocols for these materials have not been sufficiently investigated. Therefore, in this study, *C. albicans* adhering to heat-cured and additive manufactured resin specimens was removed using ozonated water, ultrasonic cleaning, and UV irradiation.

Ozone decomposes in water to produce oxygen molecules and oxygen atoms, which subsequently react with hydrogen to generate hydroxyl radicals [39,40]. These hydroxyl radicals possess strong oxidizing properties, damaging cell walls and membranes, disrupting cellular homeostasis and morphology, and exerting potent antimicrobial effects. In the present study, *C. albicans* adhering to heat-cured resin specimens was sterilized by immersion in ozonated water (1.4 ± 0.3 ppm) for 5 min, resulting in a significant reduction in the number of viable cells. Previous in vitro studies have demonstrated that ultrasonic cleaning of *C. albicans* on heat-cured resin using 4 mg/L ozonated water achieved a sterilization efficacy comparable to that of denture cleansers [34] and inhibited the growth of *C. albicans* in 11 ppm ozone ultrafine bubble water [41]. The findings of this study similarly indicated that immersion in low-concentration ozonated water for a short duration yielded viable cell counts equivalent to those obtained with denture cleansers, indicating that *C. albicans* can be effectively removed without the use of chemical agents.

Ultrasonic cleaning removes microorganisms through the cavitation effect [42]. The ultrasonic cleaning of maxillary complete dentures eliminated approximately 88.4% of *Candida* spp. [23]. In this study, ultrasonic cleaning for 5 min removed 99.1% of *C. albicans* from heat-cured resin specimens. This finding suggests that this method is effective in reducing adherent fungal cells.

Near-ultraviolet light (200~380 nm) also exhibits fungicidal activity by inducing damage to microbial nucleic acids. Binns et al. reported that UV irradiation at 254 nm serves as an effective alternative to chemical cleaning methods based on peroxide systems [35]. In the present study, the number of viable *C. albicans* counts on heat-cured resin specimens decreased to 8.3 × 10^1^ and 1.8 × 10^1^ CFU/mL following UV1 and UV3 treatments, and to 2 × 10^0^ CFU/mL following UV3 treatment on additive manufactured resin specimens. These findings suggest that UV3 irradiation exerts a potent fungicidal effect against *C. albicans*, even with short exposure durations.

Denture cleansers are typically formulated with active ingredients such as peroxides, enzymes, hypochlorous acid, or acids, each exhibiting antifungal activity against *C. albicans* through distinct mechanisms of action. In the present study, two types of denture cleansers were employed: peroxide (PO) and enzyme-based products (PK). In peroxide systems, dissolution in water generates reactive oxygen species, which produce fungicidal and bleaching effects. In enzymatic systems, fungal lytic enzymes degrade cell walls, leading to osmotic imbalance and subsequent cell lysis. Numerous studies have reported that denture cleansers are highly effective in removing *C. albicans* from denture surfaces [25].

A combination of mechanical and chemical cleaning methods is generally recommended for denture cleaning, as it enhances overall cleaning efficacy [34,43,44]. In the present study, *C. albicans* was removed from resin specimens using a combination of ozonated water immersion, ultrasonic cleaning, and UV irradiation. The results demonstrated that the number of viable *C. albicans* on the heat-cured resin specimens in the TP group (TP1, TP3, TP5) was comparable to that observed with the denture cleanser (PO, PK), whereas viable *C. albicans* counts in the TP3 group were below the detection limit. The combination of ozonated water immersion, ultrasonic cleaning, and UV irradiation was effective in eliminating *C. albicans*, likely due to the fungicidal effects of ozonated water and UV irradiation in addition to the physical removal through ultrasonic cleaning. However, a small number of viable *C. albicans* (2.0 × 10^0^ CFU/mL) was detected in the TP3 group of additive manufactured resin specimens. Previous studies have reported that *C. albicans* exhibits stronger adherence to additive manufactured resins than to resins produced by cutting or heat curing [45,46,47,48]. In the present study, resin specimens were prepared to replicate the surface roughness (Ra) of the denture mucosal surface. In clinical settings, the mucosal surfaces of dentures were not polished to avoid compromising the fit. Accordingly, both the heat-cured and additive manufactured resin specimens were ground using a carbide bar, resulting in surface roughness values of 0.65 ± 0.02 µm for the heat-cured resin specimen and 0.71 ± 0.02 µm for the additive manufactured resin specimen. The top, bottom, and side surfaces were then uniformly polished with 600-grid water-resistant abrasive paper and used as experimental specimens. The average surface roughness of the resin specimens was measured to be 0.66 ± 0.03 µm for the heat-cured acrylic resin specimens and 0.70 ± 0.02 µm for the three-dimensionally printed resin specimens. Since surface roughness was standardized through polishing, the results suggest that the polymerization method and inherent material properties influenced the results regarding the removal efficacy against *C. albicans*. Further investigation is required to clarify the influence of surface properties.

SEM observation was conducted to confirm whether *C. albicans* was removed from the resin specimens after treatment and to observe its morphology. Fluorescence imaging was used to determine the viability (live/dead status) of *C. albicans.* SEM and fluorescence observations revealed the presence of *C. albicans* in all treated samples. SEM demonstrated that *C. albicans* existed in both yeast and mycelial forms, with no distinct morphological changes associated with the different removal methods (Figure 4 and Figure 5). Fluorescence microscopy showed that nonviable *C. albicans* remained on the resin surfaces following each treatment. Accordingly, the null hypothesis was rejected, as all treatments were effective in inactivating *C. albicans*. However, complete elimination of *C. albicans* was not achieved.

The results of this study demonstrated that the combined application of the three treatments was effective in removing *C. albicans* from the resin specimens. The findings suggest three potential advantages of applying this denture cleaning method in clinical settings. First, as no chemical agents are used, the cleaning medium can be safely ingested, thereby minimizing mucosal irritation. Second, the duration of denture cleaning can be reduced, as an effective cleaning effect is achieved within a short period. Third, as the method is independent of individual brushing skills, it is suitable for older adults with diminished manual dexterity and cognitive function, as well as for caregivers who do not have expertise in denture cleaning. Therefore, we believe that developing a new denture-cleaning device that sequentially incorporates these three treatments could allow both older adults in need of care and caregivers to clean dentures more easily and conveniently.

As surface properties influence microbial adhesion, conventional denture cleaning methods may be insufficient for complete denture decontamination. In the present study, the results obtained for additive manufactured resin specimens were comparable to those for heat-cured resin specimens. As the use of technology in denture fabrication continues to expand, further investigation of cleaning methods optimized for each manufacturing technique and material property is warranted.

A limitation of this study was the use of a single *C. albicans* strain. Young et al. reported that metabolic activity and biomass increased when *C. albicans* was incorporated into a biofilm [49]. Similarly, Andrade et al. demonstrated that the use of a foaming agent significantly affected *Streptococcus mutans* but not *C. albicans* [50]. These findings suggest that future studies should consider the influence of multispecies biofilms, the variable sensitivities of different microorganisms to cleaning methods, and the removal efficacy against mature biofilms cultured for more than 24 h.

In this study, UV irradiation was applied from a single direction. As dentures possess a complex morphology, optimizing the irradiation distance and direction could further enhance the sterilization effect. Given that the wavelength and irradiation distance differed between this and previous studies, future investigations should evaluate the effects of 285 nm UV irradiation on heat-cured and additive manufactured acrylic resins. In addition, ozonated water degrades polymers by cleaving molecular main chains. Therefore, its effects on the structural integrity of heat-cured and additive manufactured acrylic resins should also be investigated. Building on these considerations, future studies will examine the effects of factors influencing the mechanical properties and chemical durability of resin materials produced by different manufacturing methods.

## 5. Conclusions

*C. albicans* adhering to the heat-cured and additive manufactured resin specimens was reduced by ozonated water, ultrasonic cleaning, or UV irradiation individually, regardless of the fabrication methods. Furthermore, the combined application of these three treatments reduced the viable *C. albicans* count to below the detection limit in the heat-cured resin specimens. Meanwhile, residual *C. albicans* remained on the surface of the heat-cured and additionally manufactured resin specimens after each treatment. The treatments for *C. albicans* on denture base resin could be useful to remove or sterilize.

## Data Availability

The data presented in this study are openly available in Iwate Medical University at https://iwatemed.repo.nii.ac.jp/.

## Abbreviation

The following abbreviation is used in this manuscript:

SEM	Scanning electron microscope
